# New Coordination Polymers of Selected Lanthanides with 1,2-Phenylenediacetate Linker: Structures, Thermal and Luminescence Properties

**DOI:** 10.3390/ma14174871

**Published:** 2021-08-27

**Authors:** Renata Łyszczek, Iwona Rusinek, Agnieszka Ostasz, Justyna Sienkiewicz-Gromiuk, Dmytro Vlasyuk, Marcin Groszek, Agnieszka Lipke, Oleksiy Pavlyuk

**Affiliations:** 1 Department of General and Coordination Chemistry and Crystallography, Institute of Chemical Sciences, Faculty of Chemistry, Maria Curie-Skłodowska University in Lublin, M.C. Skłodowskiej Sq. 2, 20-031 Lublin, Poland; iwona.rusinek@mail.umcs.pl (I.R.); agnieszka.ostasz@mail.umcs.pl (A.O.); justyna.sienkiewicz-gromiuk@mail.umcs.pl (J.S.-G.); dmytro_vlasyuk@mail.ru (D.V.); marcingroszek@interia.pl (M.G.); 2 Department of Inorganic Chemistry, Institute of Chemical Sciences, Faculty of Chemistry, Maria Curie-Skłodowska University in Lublin, M.C. Skłodowskiej Sq. 2, 20-031 Lublin, Poland; agnieszka.lipke@mail.umcs.pl; 3 Department of Inorganic Chemistry, Faculty of Chemistry, Ivan Franko National University of Lviv, Kyryla and Mefodiya St. 6, 79005 Lviv, Ukraine; pavalex@gmail.com

**Keywords:** coordination polymers, crystal structure, 1,2-phenylenediacetate linker, thermal analysis, infrared spectra, luminescence

## Abstract

Solvothermal reactions of lanthanide (III) salts with 1,2-phenylenediacetic acid in *N*,*N*′-dimethylformamide (DMF) solvent lead to the formation of the metal complexes of the general formula Ln_2_(1,2-pda)_3_(DMF)_2_, where Ln(III) = Pr(**1**), Sm(**2**), Eu(**3**), Tb(**4**), Dy(**5**), and Er(**6**), 1,2-pda = [C_6_H_4_(CH_2_COO)_2_]^2−^. The compounds were characterized by elemental analysis, powder and single-crystal X-ray diffraction methods, thermal analysis methods (TG-DSC and TG-FTIR), infrared and luminescence spectroscopy. They exhibit structural similarity in the two groups (Pr, Sm, and Eu; Tb, Dy, and Er), which was reflected in their thermal behaviours and spectroscopic properties. Single-crystal X-ray diffraction studies reveal that Sm(**2**) and Eu(**3**) complexes form 2D coordination polymers with four crystallographically independent metal centers. Every second lanthanide ion is additionally coordinated by two DMF molecules. The 1,2-phenylenediacetate linker shows different denticity being: penta- and hexadentate while carboxylate groups exhibit bidentate-bridging, bidentate-chelating, and three-dentate bridging-chelating modes. The infrared spectra reflect divergence between these two groups of complexes. The complexes of lighter lanthanides contain in the structure coordinated DMF molecules, while in the structures of heavier complexes, DMF molecules appear in the inner and outer coordination sphere. Both carboxylate groups are deprotonated and engaged in the coordination of metal centers but in different ways in such groups of complexes. In the groups, the thermal decomposition of the isostructural complexes occurs similarly. Pyrolysis of complexes takes place with the formation of such gaseous products as DMF, carbon oxides, *ortho*-xylene, ethers, water, carboxylic acids, and esters. The complexes of Eu and Tb exhibit characteristic luminescence in the VIS region, while the erbium complex emits NIR wavelength.

## 1. Introduction

**Figure 1:**
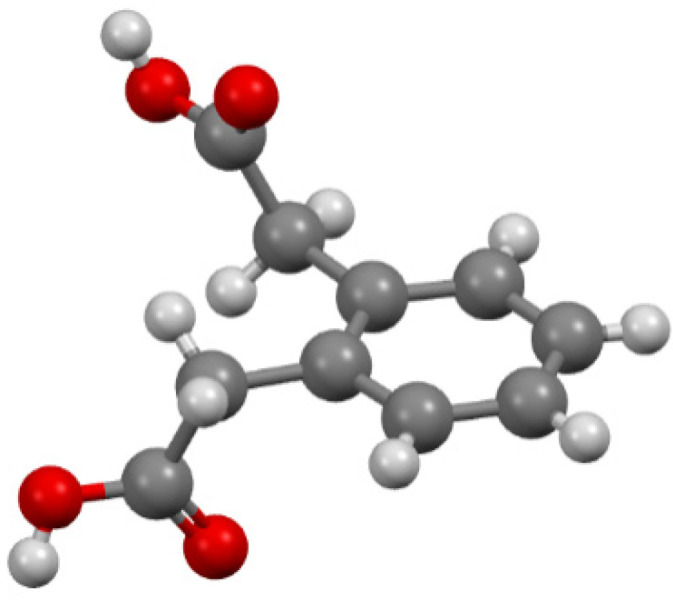
Scheme of 1,2-phenylenediacetic acid.

As an important branch in the field of supramolecular chemistry and crystal engineering, the design and assembly of metal–organic coordination frameworks (MOFs) or coordination polymers (CPs) have stimulated the interest of chemists over the past few decades [1,2,3,4,5,6,7], not only due to their intriguing network topologies, but also the possible application. Lanthanide coordination polymers (CPs) have been studied due to their applicability in gas storage, catalysis, and luminescence [8,9,10,11,12,13,14,15]. To date, most of the organic ligands used in MOFs chemistry are limited to a rigid aromatic carboxylate containing ligand [16,17,18], whereas the role of a flexible carboxylate ligand is somewhat ignored. Compared with rigid ligands, using flexible ones to construct coordination polymers seem more difficult and developing systematic methodologies of synthesis materials from prior design structure via flexible ligands are still a great challenge, which may be attributed to their unpredicted structures. However, flexible molecules can easily adjust their conformations to meet the coordination requirement of the metal. The 1,2-phenylenediacetic acid (1,2-H_2_pda) has flexible –CH_2_– groups that cut conjugation and allow free rotation of the carboxylate groups (Figure 1). This dicarboxylic acid bears two C atoms in the aliphatic side-chains of a benzene ring. The acetic moieties are twisted away from the plane of the phenylene unit due to sp^3^ hybridization of methylene carbon atoms. Some coordination polymers based on 1,2-phenylenediacetic ligand, often together with another rigid ligand, have been reported with transition metal ions [19,20,21,22,23,24]. To the best of our knowledge, there are only nine crystal structures of lanthanide coordination polymers that involve only this ligand as confirmed by the CSD [25] data search. These crystal structures, that correspond to compounds with chemical formula {[Ln_2_(1,2-pda)_3_(H_2_O)_x_]·y(H_2_O)}_n_ have been reported with different lanthanide ions: La(III) [25,26], Nd(III), Dy(III), Er(III) [25], Tb(III), Ho(III) [27], and Gd(III) [28].

The solvent plays a crucial role in the synthesis of coordination polymers (CPs). In fact, the predictability of the final network can be a challenge since it is a consequence of the self-assembling process that involves competing, reversible and simultaneous interactions among the metal, ligand, counterion, and solvent. The final coordination outcome is not easy to control, both in terms of topology and network dimensionality. The interaction energy between the metal and ligand decreases, the system is more prone to be affected by other parameters, such as the solvent. The coordinating solvent can block a different number of coordination sites, leading to different CP architectures.

The carboxylate ligands have attracted increasing attention in the construction of CPs [29,30,31]. This is due to the fact that the carboxylate ligands have rich coordination modes [32] and are susceptible to the reaction conditions. Through controlling the reaction conditions, carboxylate CPs with diverse structures can be synthesized. Recently, luminescent CPs as efficient sensing materials have attracted great attention for their tunable structures and good optical properties [33,34,35,36,37,38].

Previously, we have focused on the hydrothermal synthesis and characterization of lanthanide complexes based on the 1,3-phenylenediacetate ligand [39]. As the continuation of our investigations on lanthanide carboxylates [40,41,42,43,44,45], six novel coordination polymers of selected lanthanide ions (i.e., Pr(III), Sm(III), Eu(III), Tb(III), Dy(III), and Er(III)) with flexible 1,2-phenylenediacetate linker were synthesized and characterized. The compounds were prepared by the solvothermal approach from the *N*,*N*′-dimethylformamide medium. Their structures were determined based on the elemental analysis, X-ray diffraction methods, and infrared spectroscopy. The TG-DSC and TG-FTIR methods in air and nitrogen were utilized for determination of their thermal behaviour. The luminescence properties of europium, terbium, and erbium complexes were investigated at room temperature.

## 2. Materials and Methods

### 2.1. Synthesis of Coordination Polymers

Commercial reagents were purchased from Sigma-Aldrich (ACS grade) and used as received.

The complexes **1**–**6** were obtained in the reaction of 1 mmol of lanthanide salt with 1.5 mmol of 1,2-phenylenediacetic acid (C_6_H_4_(CH_2_CO_2_H)_2_ under solvothermal conditions. The stechiometric amounts of lanthanide(III) nitrates (Pr(NO_3_)_3_·xH_2_O—0.3269 g; Tb(NO_3_)_3_·xH_2_O—0.4350 g; Dy(NO_3_)_3_·xH_2_O—0.4386 g) or lanthanide(III) chlorides (SmCl_3_·xH_2_O—0.2567 g; EuCl_3_·xH_2_O—0.3664 g; and Er(NO_3_)_3_·xH_2_O—0.2736 g) were deliquesced in 10 mL of *N*,*N*′-dimethylformamide (C_3_H_7_NO). The 1,2-phenylenediacetic acid (0.2913 g) was converted into a solution by adding 20 mL of *N*,*N*′-dimethylformamide. Subsequently, solutions of lanthanide salt and organic linker were combined, no precipitations were observed. Then, the obtained mixtures were placed in a Teflon-lined stainless steel autoclave, deposited in a laboratory dryer and heated at 140 °C for 72 h under autogenous pressure. After cooling to room temperature, the products were filtered off, washed with DMF and dried at room temperature. For complexes of Sm and Eu, suitable crystals for single-crystals X-ray analysis were isolated from the resulted suspensions before filtration. The yield of synthesis of complexes based on the lanthanide(III) ion was in the range of 67–78%. Elemental analysis: For **1** (Pr_2_C_36_H_38_N_2_O_14_) Calcd (%): C, 43.00; H, 3.88; N, 2.78. Found: C, 42.59; H, 3.46; N, 2.56; for **2** (Sm_2_C_36_H_38_N_2_O_14_) Calcd (%): C, 42.21; H, 3.81; N, 2.73. Found: C, 41.67; H, 3.66; N, 2.54; for **3** (Eu_2_C_36_H_38_N_2_O_14_) Calcd (%): C, 42.07; H, 3.79; N, 2.72. Found: C, 42.25; H, 3.59; N, 2.59; for **4** (Tb_2_C_36_H_38_N_2_O_14_) Calcd (%): C, 41.51; H, 3.74; N, 2.69. Found: C, 40.89; H, 3.22; N, 2.63; for **5** (Dy_2_C_36_H_38_N_2_O_14_) Calcd (%): C, 41.23; H, 3.72; N, 2.67. Found: C, 41.03; H, 3.69; N, 2.42; for **6** (Er_2_C_36_H_38_N_2_O_14_) Calcd (%): C, 40.86; H, 3.68; N, 2.64. Found: C, 40.51; H, 3.24; N, 2.33.

### 2.2. Methods

The IR spectra of acid and the prepared complexes were recorded in the range 4000–400 cm^−1^ by means of Specord M80 (Carl Zeiss Jena, Oberkochen, Germany) spectrophotometer using the KBr pellet technique.

The C, H, and N analyses were carried out with an EuroEA3000 elemental analyzer (EuroVector S.p.A., Milan, Italy).

The powder X-ray diffraction experiments on the bulk materials were performed on a PANalytical Empyrean (Panalytical, Almelo, The Netherlands) automated diffractometer (Bragg-Brentano method; *Cu*-K_α_ radiation) via continuous scan with a step size of 0.02626° over the scattering angular range 2*θ* between 5 and 90° at ambient temperature. Indexation of the recorded diffraction profiles and calculations of unit cell parameters were carried out using the DICVOL06 [46] program as implemented in the FullProf Suite 2.05 (Laboratoire Léon Brillouin (CEA-CNRS) CEA/Saclay, Gif sur Yvette Cedex, France) package [47]. Additionally, the reliability of the calculated unit cells was assessed by the figures of merit M(20) [48] and F(20) [49]. In the case of Sm (**2**) and Eu (**3**) complexes, the experimental powder X-ray diffraction patterns were collated with those simulated by the Mercury 4.3.1 (The Cambridge Crystallographic Data Centre (CCDC), Cambridge, UK) software [50] generated based on single-crystal X-ray data.

Thermal properties of the as-synthesized complexes were investigated employing the thermogravimetric analysis coupled with differential scanning calorimetry (DSC) using a SETSYS 16/18 (Setaram, Caluire, France) thermal analyzer. The measurements were made in the temperature range 30–1000 °C at a heating rate of 10 °C min^−1^ under the dynamic air atmosphere (v = 0.75 dm^3^ h^−1^). The samples of complexes (6–8 mg) were heated in the alumina crucibles.

The Fourier transform infrared spectroscopy (FTIR) spectra of gaseous products of thermal decomposition of investigated compounds were measured using a Q5000 TA apparatus (TA Instruments, New Castle, DE, USA) coupled with the Nicolet 6700 FTIR spectrophotometer (Thermo Scientific, Waltham, MA, USA). The samples (~20 mg) were heated in open platinum crucibles up to 700 °C at a heating rate of 20 °C min^−1^ in a flowing nitrogen atmosphere (25 cm^3^ min^−1^).

Luminescence excitation and emission spectra were carried out at room temperature on a QuantaMaster™ spectrofluorometer (Photon Technology International, Birmingham, United Kingdom) equipped with a continuous 75 W Xe-arc lamp as the light source. The spectra were corrected with respect to the source and detector.

### 2.3. Single-Crystal Structure Determination and Refinement

Unit cell determination and data collection of the **2**–**3** compounds were performed at 293 K on an Oxford Diffraction Xcalibur CCD diffractometer (Oxford Diffraction Ltd., Abingdon, UK) with the graphite-monochromated MoK*α*-radiation (λ = 0.71073 Å). The programs CrysAlis CCD and CrysAlis Red [51] were used for data collection, cell refinement, and data reduction. A multi-scan absorption correction has been applied. The structures **2**–**3** were solved by direct methods using SHELXS-97 and refined by the full-matrix least-squares on F^2^ using SHELXL-97 [52] and OLEX2-1.5 (OlexSys Ltd., Durham, England) software [53]. All non-hydrogen atoms in **2** have been refined anisotropically, hydrogen atoms in metal-organic moieties were placed in a calculated position and refined in rigid mode. The structures were verified using the ADDSYM algorithm from the program PLATON [54] and no higher symmetries were found. Crystallographic and experimental details for **2** and **3** complexes are summarized in Table 1.

CCDC 2099146 [for **2**] and 2099147 [for **3**] contain the supplementary crystallographic data for this paper. These data can be obtained free of charge from the Cambridge Crystallographic Data Centre.

## 3. Results and Discussion

We have synthesized six novel lanthanide(III) complexes with 1,2-phenylenediacetic acid of the general formula: Ln_2_(1,2-pda)_3_(DMF)_2_; where Ln(III) = Pr(**1**), Sm(**2**), Eu(**3**), Tb(**4**), Dy(**5**), and Er(**6**); 1,2-pda = [C_6_H_4_(CH_2_COO)_2_]^2−^ under solvothermal conditions in the *N*,*N’*-dimethylformamide solution. All compounds were obtained in the form of polycrystalline powdered samples but in the case of Sm and Eu complexes, we have been able to isolate crystals suitable for further single-crystal X-ray diffraction analysis.

### 3.1. Powder X-ray Diffraction Studies

**Figure 2:**
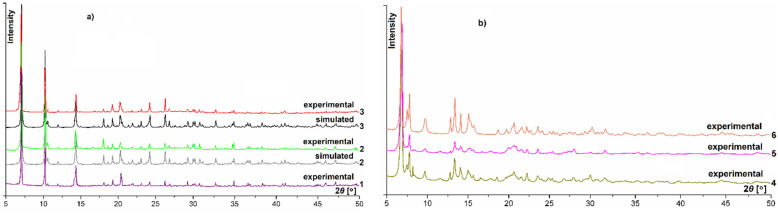
Powder profiles for orthorhombic (**a**) and monoclinic (**b**) complexes over the 2*θ* angle range from 5 to 50°.

The phase purity and crystalline nature of the bulk samples in the solid-state were verified based on powder X-ray diffraction measurements. Furthermore, the unit cell dimensions of the polycrystalline materials were also calculated through powder patterns indexing. The compounds in question form two isostructural series. The first one consists of light lanthanide compounds **1**–**3** crystallizing in the orthorhombic system, whereas the second one contains heavy lanthanide complexes **4**–**6** which, in turn, exhibit a lower monoclinic symmetry. The unit cell parameters calculated for polycrystalline materials are listed in Table 2, while the well-defined Bragg’s peaks at particular 2*θ* angles limited to 50° of both series are presented in Figure 2.

The two different patterns observed in the recorded powder profiles also clearly indicate the formation of two different crystalline forms of investigated samples, which is consistent with the obtained two sets of solutions regarding the unit cells dimensions.

Additionally, in the event of Sm (**2**) and Eu (**3**) complexes, both patterns fit very well with each other, which confirms that the studied materials represent a pure phase with good crystallinity (Figure 2a). Moreover, the noticeable compliance of the unit cell parameters obtained from both powder and single-crystal X-ray diffraction analyses also reflect the phase purity of the studied coordination products.

### 3.2. Crystal Structure Description for **2** and **3** Complexes

The 1,2-phenylenediacetic acid form with the Sm(III) and Eu(III) ions isostructural complexes [Sm_2_(1,2-pda*)*_3_DMF)_2_]_n_ and [Eu_2_(1,2-pda*)*_3_(DMF)_2_]_n_ crystallize in the orthorhombic *Pca*2_1_ space group (Table 1). These lanthanide complexes are the first examples of anhydrous lanthanide coordination polymers with such ligand. In previously reported structures, one [25,27,28] or two [26] aqua ligands have appeared in the inner coordination sphere of lanthanide centers. Additionally, lattice water molecules have occupied the free space in the three-dimensional frameworks of hydrated complexes.

**Figure 3:**
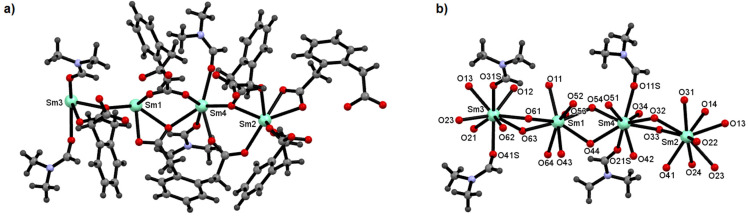
(**a**) Asymmetric unit of complex **2**. (**b**) Coordination environment of samarium atoms in **2** with emphasized DMF molecules.

We have discussed peculiarities of the crystal structure of the first compound as a representative. The asymmetric unit contains four symmetrically independent samarium(III) ions, six 1,2-phenylenediacetate and four *N*,*N*′-dimethylformamide molecules (Figure 3a). The metal atoms in the crystal structure of the samarium complex possess a nine-vertex coordination environment. The coordination spheres of Sm1 and Sm2 atoms consist of only carboxylate oxygen atoms from organic ligands, while the atoms Sm3 and Sm4 are coordinated by seven carboxylate oxygen atoms and two oxygen atoms from DMF molecules (Figure 3b). All metal centers are bonded by six different 1,2-phenylenediacetate moieties. The bond lengths Sm-O_carb._ range from 2.325(7) to 2.739(7) Å, while Sm-O_DMF_ bond lengths vary from 2.413(7) to 2.466(7) Å (Supplementary Materials, Table S1). These values are in good agreement with those reported for other samarium(III) carboxylates as witnessed by a search in the Cambridge Structural Database [25]. The remaining bond lengths and angles in the molecules of 1,2-phenylenediacetate molecules are within normal ranges (Table S2).

**Figure 4:**
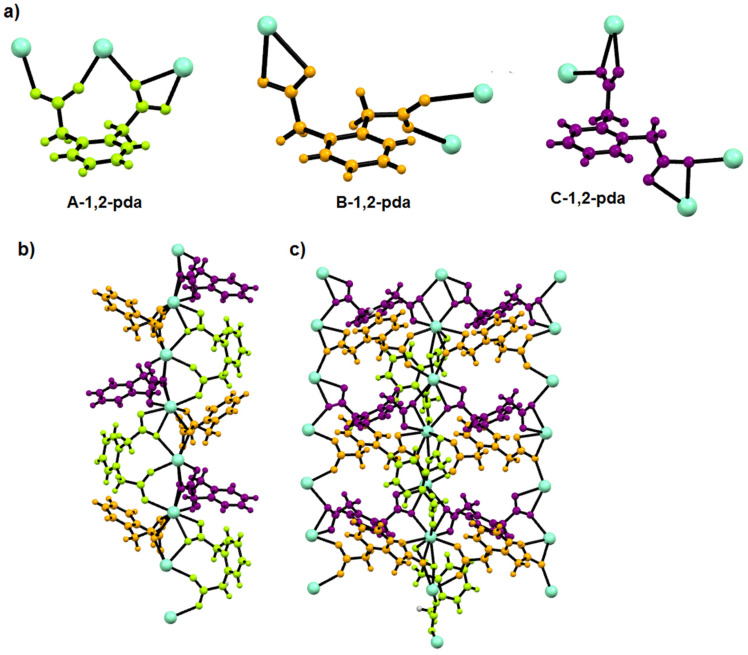
(**a**) Conformation and coordination modes of 1,2-pda ligand. (**b**) Representation of different functions of 1,2-pda ligand in the **2** in view along the *a* axis. (**c**) View of **2** in the *ac* plane. DMF molecules were omitted for clarity.

In the structure of the Sm complex, three different conformations and coordination modes of 1,2-pda ligand can be distinguished (Figure 4a). In molecule A-1,2-pda, neighbouring carboxylate groups from acetate arms are situated in the *cis* position, being on the same side of the plane defined by the phenylene ring. Carboxylate groups exhibit bidentate-bridging (μ_2_-η^1^:η^1^) and three dentate bridging-chelating (μ_2_-η^1^:η^2^) characters. This linker bonds three neighbouring lanthanide centers, exhibiting a pentadentate character. The B-1,2-pda linker behaves as a tetradentate ligand but –COO groups are located on the opposite side of the phenylene ring in the *trans* fashion. The carboxylate groups exhibit bidentate-chelating and bidentate-bridging characters. The *trans* arrangement of carboxylate groups is also observed in the C-1,2-pda ligand, but both –COO groups adopt bridging-chelating (μ_2_-η^1^:η^2^) modes. The C-1,2-pda molecule can be regarded as a hexadentate ligand. Taking into account the geometry and coordination abilities of such ligands, they play a different role in the structure (Figure 4b).

The A-1,2-pda molecules with *cis* conformation of COO groups take part in the connection of lanthanide ions into the linear chains of metal centers in the *c* direction. Such type of lanthanide(III) coordination through the 1,2-phenylenediacetate linker has not been reported yet [25]. In the known crystal structures of lanthanide 1,2-phenylenediacetates, organic ligands always coordinated metal centers from different Ln chains. The metal centers are additionally bonded via B-1,2-pda and C-1,2-pda molecules. The adjacent lanthanide centers are joined by one μ_2_-η^1^:η^1^ and two μ_2_-η^1^:η^2^ carboxylate groups. The B-1,2-pda and C-1,2-pda ligands connect lanthanide chains into the two-dimensional network extended in the *ac* plane (Figure 4c).

Considering the presence of only carbon atoms in the structure of compounds as potential electron density donors and the realization of only the acceptor function of oxygen atoms in the formation of hydrogen contacts, the effect of hydrogen bonding on the structure of the compound is predictably small [55,56]. The weak hydrogen contacts C–H···O were observed only in the metal-organic layers area (Table S3). The previously reported lanthanide complexes with 1,2-phenylenediacetate linker form three-dimensional coordination polymers [25,26,27,28].

### 3.3. Infrared Spectra of Metal Complexes

**Figure 5:**
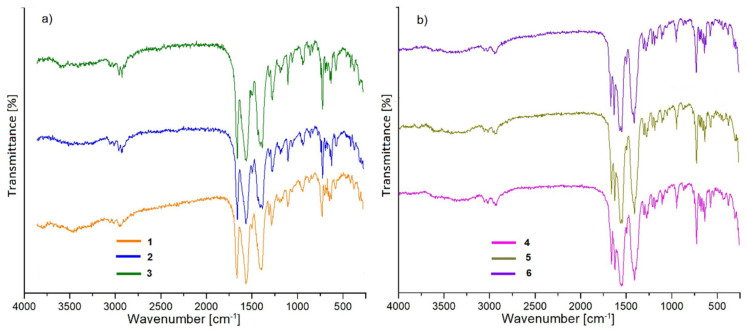
Infrared spectra of isostructural complexes: (**a**) **1**–**3**; (**b**) **4**–**6**.

Analysis of the infrared spectra of the synthesized complexes enabled the determination of types of ligands bonded with metal centers. Based on the comparison of the IR spectra of metal complexes with the spectrum of free 1,2-phenylenediacetic (Figure S1) acid, it can be concluded that both carboxylic groups were deprotonated and transferred into the carboxylate ones (Figure 5). Taking account of the fact of the great affinity of oxygen atoms to the lanthanide ions, the formation of Ln-O_carb._ bonds can be concluded, which was reflected in the presence of carboxylate groups vibrations in the infrared spectra of metal complexes. The IR spectrum of free 1,2-pda acid is dominated by a very strong band at 1692 cm^−1^ attributed to the stretching ν(C=O) vibrations as well as strong bands at 1256 and 928 cm^−1^ from the stretching ν(C–O) and deformation β(OH) vibrations of COOH groups [57,58]. These bands vanished in the spectra of complexes and are replaced by the asymmetric (ν_asym_) and symmetric (ν_sym_) stretching modes of carboxylate groups. For the complexes of Pr, Sm, and Eu, the bands from ν_asym_ vibrations appear at 1560, 1564 and 1560 cm^−1^, respectively, while the band derived from the ν_sym_ vibrations are observed at 1388, 1384 and 1384 cm^−1^ (Figure 5a). On the other hand, positions of stretching carboxylate vibrations in the complexes of Tb, Dy, and Er are slightly different. The bands from the asymmetric stretching vibrations are split and the maxima of peaks are observed at 1556/1548; 1560/1548, and 1572/1548 cm^−1^ for Tb, Dy, and Er complexes, respectively (Figure 5b). The bands assigned to the symmetric (ν_sym_) stretching vibrations appear in all complexes at 1412 cm^−1^. These observations are consistent with the statement that coordination modes of COO groups in these two groups of complexes are different. In the complexes of Pr, Sm, and Eu, carboxylate groups bind Ln(III) ions in the bidentate-bridging and three-dentate bridging–chelating fashion, while for the remaining compounds other coordination modes can be observed.

The presence of DMF molecules in the structures of the complexes is also clearly reflected in the infrared spectra. The all spectra are characterized by relatively weak bands in the range number 3100–2800 cm^−1^, which correspond to the stretching vibrations of methylene groups from 1,2-phenylenediacete ligands and methylene groups from *N*,*N*′-dimethylformamide molecules. In the first group of compounds (Pr, Sm, and Eu), stretching vibrations of carbonyl groups from DMF molecules coordinated to the lanthanide center give bands at 1660–1656 cm^−1^.

For the remaining complexes, their infrared spectra show two well-separated bands at 1668 and 1628 cm^−1^ ascribed to the ν(C=O) group of non-equivalent DMF molecules [59]. It is reasonable to suggest that DMF molecules are bonded in the structure differently. Solvent molecules are most probably coordinated with lanthanide centers, as well as located in the channels/free spaces in the structure of complexes.

### 3.4. Thermal Analysis in Air and Nitrogen Atmosphere

**Figure 6:**
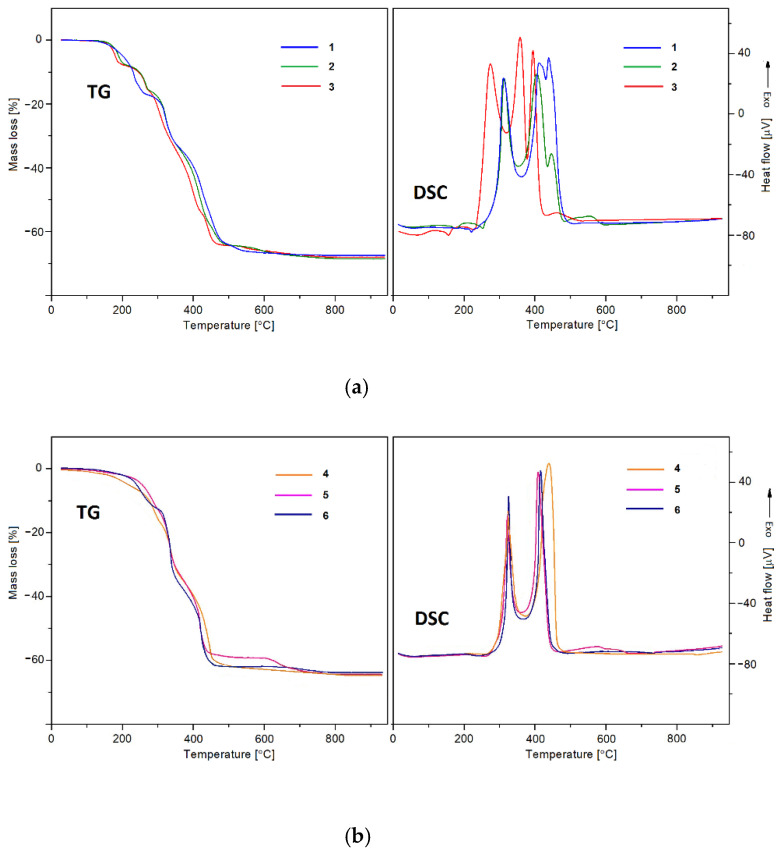
(**a**) TG and DSC curves of complexes **1**–**3** in air. (**b**) TG and DSC curves of complexes **4**–**6** in air.

The thermal stability of the synthesized complexes in the air atmosphere was examined by means of thermogravimetry (TG) and differential scanning calorimetry (DSC). The gaseous products of their thermal decomposition were identified based on the FTIR spectra recorded along with the TG curves during heating in nitrogen. As can be seen from the data of thermal analysis given in Table 3, the complexes **1**–**3** exhibit higher thermal stability in comparison to compounds **4**–**6** (Figure 6).

The compounds of lighter lanthanides **1**–**3** are stable up to 120, 138, and 146 °C, respectively, while complexes **4**–**6** are stable at room temperature but their heating results in the desolvation process which takes place above 35, 40 and 80 °C, respectively (Figure 6a). The removal of DMF molecules is observed in the two well-separated stages only in the complexes of Eu and Sm, while for the remaining compounds the solvent is released in one step up to 352 °C. For complexes **2** and **3**, the first molecule of DMF is liberated up to about 220 °C, while the second one is up to 292 and 346 °C, respectively. As can be seen from the DSC curves of complexes **2** and **3**, the release of the first DMF molecule is accompanied by an endothermic effect with a peak top at 201 and 187 °C, respectively. The second endothermic effect at about 278 °C overlaps with the exothermic effect connected with the burning of organic ligand (Figure 6b).

These observations point out the fact that the release of the second DMF molecule causes degradation of inorganic-organic frameworks (Figure 6a). In the case of complexes **4**–**6**, the loss of DMF molecules takes place without easily detectable energetic effects on the DSC curves (Figure 6b). At higher temperatures, the decomposition process of unstable products occurs along with the burning of organic ligands. The final solid products of decomposition, i.e., suitable lanthanide oxides (Pr_6_O_11_, Eu_2_O_3_, Sm_2_O_3_, Tb_4_O_7_, Dy_2_O_3_, and Er_2_O_3_) are formed in the range 665–930 °C. The shape of the TG curve of complex **5** indicates that its decomposition proceeds with the formation of the solid intermediate Dy_2_O(CO_3_)_2_ [59]. The final mass losses in the **1**–**6** metal complexes connected with their heating in the air are in the range of 62.56–66.96%.

Regarding the pathways of thermal decomposition of the complex under consideration, it is clearly seen that the liberation of DMF molecules from the structure of the investigated complex results in the degradation of the metal-ligand framework.

**Figure 7:**
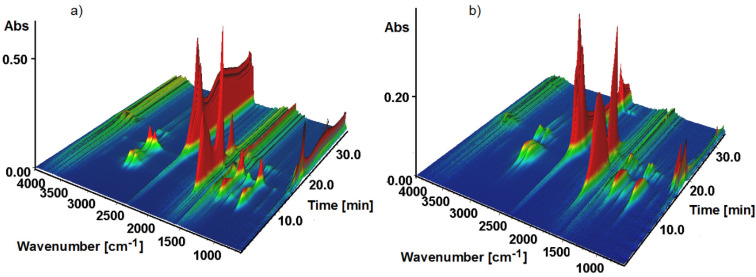
(**a**) Stacked plot of FTIR spectra of the evolved gases for **2**. (**b**) Stacked plot of FTIR spectra of the evolved gases for **6**.

**Figure 8:**
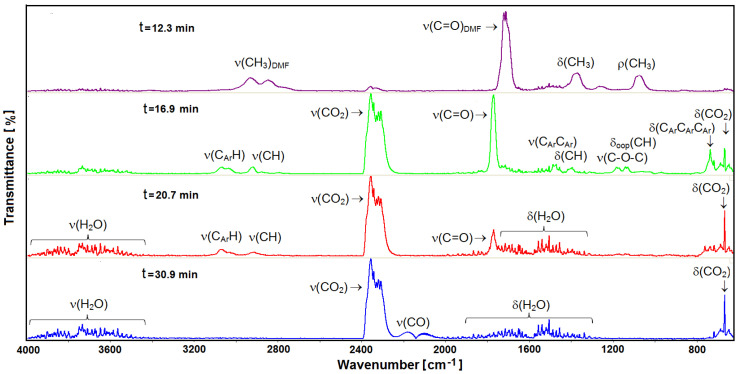
The FTIR spectra of gases evolved at different temperatures for **2**.

Thermogravimetry (TG) combined with infrared spectroscopy (FTIR) are the perfect tools for the identification of gaseous products evolved during the controllable heating of investigated materials. The complexes of samarium (**2**) and erbium (**6**) were taken as representative of light and heavy lanthanide groups of metal complexes (Figure 7). The selected FTIR spectra of volatile products of complex 2 decompositions recorded at different temperatures are given in Figure 8.

Complex **2** is stable in nitrogen atmosphere up to 157 °C (6.5 min). At higher temperatures, the liberation of DMF molecules from the structure of the metal complex takes place in several overlapping steps in the range of 6.06–16.00 min. The infrared spectra exhibit two distinct medium-strong bands occurring at 2938 and 2848 cm^−1^. These bands derived from the stretching asymmetric (ν_asym_) and symmetric (ν_sym_) vibrations of methyl groups CH_3_ from DMF. The presence of a very strong band at 1723 cm^−1^ results from the stretching vibration (ν) of carbonyl group (C=O) group of DMF. The asymmetric and symmetric bending vibrations (δ) of methyl groups (CH_3_) appear at 1457 and 1374 cm^−1^, respectively. Additionally, the relatively intense bands at 1270 and 1076 cm^−1^ can be ascribed to the rocking modes (ρ) of methyl bands derived from the gem (CH_3_)_2_N group of DMF [59,60]. Removal of *N*,*N’*-dimethylformamide molecules is observed up to about 300 °C (13.7 min). At higher temperatures, pyrolysis of the desolvated form of the complex takes place. In the temperature range of 310–400 °C (14–18 min), the intensity of different moieties emanation is the highest. The FTIR spectra show a very strong band consisting of many overlapping peaks in the wavenumber range of 2370–2300 cm^−1^ as well as those at 668 cm^−1^ due to stretching and deformation vibrations of CO_2_. Apart from carbon dioxide, the analysis of the FTIR spectra allows finding several other volatile compounds such as *ortho*-xylene, aliphatic ethers, and aliphatic acids. The identification of such compounds was made based on the presence of characteristic bands in the wavenumber ranges: 3100–2800; 1800–1600; 1200–1100; and 780–600 cm^−1^. The weak bands at 3076 and 3038 cm^−1^ derived from aromatic C-H stretching vibrations, while maxima at 2938 and 2848 cm^−1^ can be assigned to the stretching vibrations of C_Ar_H groups from phenylene ring and asymmetric and symmetric stretching modes of CH_3_ groups from o-xylene molecules. The bands from stretching vibrations of aromatic C_Ar_C_Ar_ ring appear at 1540, 1507 and 1473 cm^−1^. In-plane and out-of-plane bending vibrations δ_oop_(CH) of ortho-substituted benzene ring from CH and C_Ar_C_Ar_C_Ar_ moieties were shown at 1132, 737 and 719 cm^−1^, respectively [57,61]. Along with o-xylene molecules, also some aliphatic ethers were evolved due to bands in the wavenumber range 3000–2800 cm^−1^ and those at 1457, 1184 and 1102 cm^−1^ as a result of stretching and deformation vibrations of CH_3_ groups and stretching mode of C–O–C from ethers [62]. The FTIR spectra recorded above 340 °C displays a relatively strong band at 1772 cm^−1^, which can be assigned to the stretching vibrations of carbonyl groups most probably from some carboxylic acid or esters [62]. Further heating results in the evolution of carbon monoxide, as can be deduced based on the very diagnostic double bands with maxima at 2185 and 2107 cm^−1^ and water molecules [63]. Water molecules and carbon oxides are observed up to 700 °C.

Taking into account the thermal stability of complex **6** in the nitrogen atmosphere, we can conclude on its higher thermal stability in comparison to air. The erbium complex is stable to 150 °C (6.1 min) and then one-stage desolvation takes place to 340 °C. The highest intensity evolution of DMF molecules is observed at about 275 °C. Next, similarly as it was observed in the **2** complex, decomposition of the desolvated form of the complex is observed. The most intense bands derived from carbon dioxide, ortho-xylene, carboxylic acids, and ethers are released at about 340 °C. Intensities of stretching vibrations of carbonyl groups and carbon dioxide recorded at 17.2 min are very similar that point out the significant participation of carbonyl compounds in gaseous products of erbium complex decomposition. At higher temperatures, the evolution of carbon monoxide and water molecules is also observed.

Comparing the FTIR spectra of gaseous products of **2** and **6,** it is clearly seen that the mechanism of their degradation is different due to their different crystal structures.

### 3.5. Luminescence Investigations

**Figure 9:**
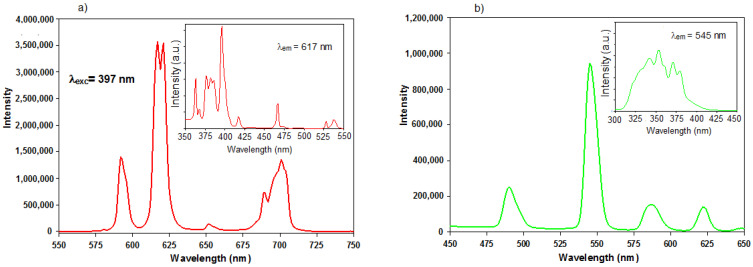
The emission luminescence spectra for complexes **3** (**a**) and **4** (**b**). The excitation spectra of complexes were inserted in the inset of emission spectra.

The excitation and emission spectra of complexes **3**, **4** and **6** were recorded in a solid state at room temperature. The excitation spectrum of **3** was obtained by monitoring the emission of Eu(III) ions at 617 nm. The excitation spectrum of complex **3** is dominated by the bands from f-f transitions of Eu(III) ions (Figure 9a inset). The bands observed at: 363, 376–386, 397, 417, 467, 528 and 538 nm were assigned to the ^7^F_0_→^5^D_4_, ^7^F_0_→^5^G_J_, ^7^F_0_→^5^L_6_, ^7^F_1_→^5^D_3_, ^7^F_0_→^5^D_2_ and ^7^F_0_→^5^D_1_ transitions [64]. The emission spectrum of Eu_2_(1,2-pda)_3_(DMF)_2_ complex was recorded after excitation with 397 nm. The spectrum shown in Figure 9a exhibits bands at 592, 617, 621, 652, 689 and 701 nm related to the emanation from the nondegenerate ^5^D_0_ excited state to the J levels of the ground term ^7^F of europium(III) ions [65]. The ^5^D_0_→^7^F_2_ transitions located at 617 and 621 nm are responsible for the typical red luminescence observed in europium(III) compounds [65].

The excitation spectrum of complex **4** was recorded by monitoring the emission of Tb(III) ion at 545 nm. The excitation spectrum of complex **4** exhibits broadband in the range of 300–425 nm, which can be attributed to the weak S_0_→S_1_ transitions of coordinated organic ligand. This spectrum shows also the strong bands at 343, 351, 370 and 380 nm arising from f-f transitions of Tb(III) ions from the ground state ^7^F_5_ to the following levels: ^5^L_6_, ^5^L_9_, ^5^L_10_ and ^5^D_3_, respectively (Figure 9b inset). These bands are very intense, which is characteristic of the weak sensitization “force” of ligand [66]. The emission spectrum of Tb_2_(1,2-pda)_3_(DMF)_2_ displays the emission bands at 490, 545, 587 and 622 nm, which were assigned to the transitions of the Tb(III) ions from the excited state ^5^D_4_ to the states ^7^F_6-4_ (Figure 9b). The most intense emission band at 545 nm corresponds to the ^5^D_4_→^7^F_5_ transition and is responsible for the green emission of such complex [67].

**Figure 10:**
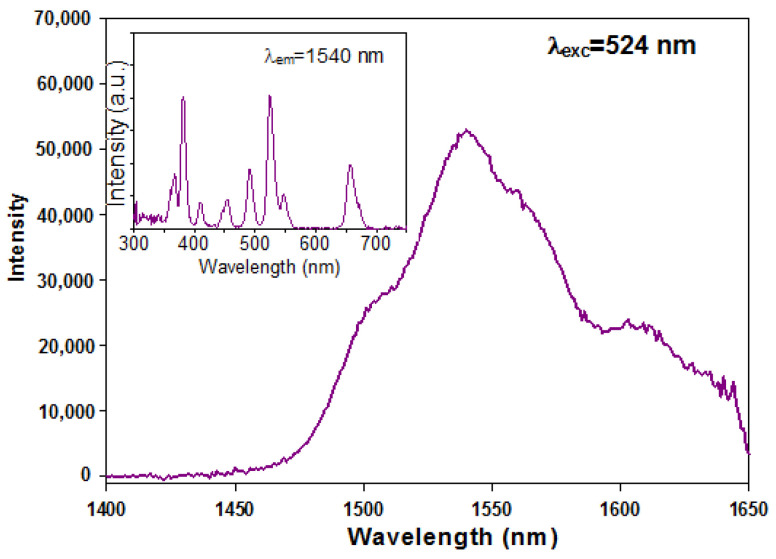
The emission and excitation spectra (inset) for complex **6**.

The excitation spectrum of complex **6** monitored at 1540 nm shows several peaks centered at: 368, 381, 410, 454, 491, 524, 547 and 657 nm originated from the f-f transitions of Er^3+^ ion from the ^4^I_15/2_ ground state to the excited levels: ^2^G_9/2_, ^2^G_11/2_, ^2^H_9/2_, ^4^F_3/2_, ^4^F_5/2_, ^4^F_7/2_, ^2^H_11/2_, ^3^S_3/2_, and ^4^F_9/2_ [68]. The profile of the excitation spectrum of complex 6 is indicative of ineffective energy transfer from the organic ligand to the metal center (Figure 10 inset).

Under excitation with λ_exc_ = 524 nm, the emission spectrum of erbium(III) complex shows broadband in NIR spectral region (Figure 10) with the maximum at 1540 nm assigned to the ^4^I_13/2_→^4^I_15/2_ transition of Er(III) ions [69].

## 4. Conclusions

Six novel crystalline lanthanide(III) complexes distinguished by phase purity have been successfully constructed with the flexible 1,2-phenylenediacetate ligand and DMF as solvent. The powder X-ray, spectral, and thermal data confirm that the obtained materials belong to two distinct isostructural groups formed by light and heavy lanthanide(III) ions, respectively. The complexes synthesized with the participation of light lanthanide(III) metal centers (Pr (**1**), Sm (**2**) and Eu (**3**)) are orthorhombic, whereas the heavy ones (Tb (**4**), Dy (**5**) and Er (**6**)) exhibit monoclinic symmetry. The crystal structures of complexes **2** and **3** are the first examples of two-dimensional coordination polymers constructed from the 1,2-phenylenediacetate linker, in which DMF molecules are coordinated with metal centers. The analysis of crystallographic data of **2** and **3** reveals the various coordination modes of carboxylate groups originated from three different conformations of 1,2-phenylenediacatate molecules. The molecules of the organic linker that adopt *cis*-conformation are predominantly responsible for generating 1D linear chains, which are further conjoined by means of two other types of 1,2-pda molecules with *trans*-conformations into 2D layered metal-organic networks. The complexes **1**–**3** exhibit higher thermal stability in comparison to the remaining complexes. The investigated complexes decompose in a multi-step fashion with evolution of DMF, carbon oxides, ortho-xylene, carboxylic acids, and ethers during heating in nitrogen. The europium and terbium complexes exhibit characteristic red and green metal-based luminescence, while the erbium complex emits in the NIR region.

Taking into account the sharp emissions of the europium(III) and terbium(III) complexes in the visible light range, these compounds can be further investigated for their potential applications in solid state lighting and display areas [70,71]. Generally, metal complexes offer better compatibility with organic matrices in the OLEDs system compared with inorganic compounds. On the other hand, the utilization of lanthanide complexes in the fabrication of OLEDs is strongly limited by their poor solubility and charge transportation features [71,72]. The other fields of their potential application is sensing of small molecules, cations and anions, pH or temperature [33]. These lanthanide complexes can be also regarded as luminescent dopants for different types of matrices, enriching in this way the group of hybrid optics materials [45,65,72].

## Figures and Tables

**Table 1:** Crystallographic data for complexes **2** and **3**.

Compound	2	3
Empirical formula	Sm_2_C_36_H_38_N_2_O_14_	Eu_2_C_36_H_38_N_2_O_14_
Formula weight	1023.38	1026.6
Crystal system	orthorhombic	
Space group	*Pca*2_1_	
*T* (K)	293	
*a* (Å)	9.2162(3)	9.2186(3)
*b* (Å)	24.8911(6)	24.9274(8)
*c* (Å)	32.4352(8)	32.3941(12)
*Z*	8	
*V* (Å^3^)	7440.7(4)	7444.0(4)
*D*_calc_ (Mg∙m^−3^)	1.827	1.832
*F(000)*	4032	4048
Measured/used reflections/refined parameters	65,341/19,700/982	68,483/19,805/982
*R_int_*	0.062	0.070
Theta range (°)	2.7–30.6	2.5–31.0
Goodness-of-fit on *F^2^*	1.05	1.02
Final *R* indices [*I* > 2σ(*I*)]	*R*_1_ = 0.046, *wR*_2_ = 0.090	*R*_1_ = 0.047, *wR*_2_ = 0.071
*R* indices (all data)	*R*_1_ = 0.070, *wR*_2_ = 0.081	*R*_1_ = 0.073, *wR*_2_ = 0.081

**Table 2:** Lattice parameters for polycrystalline forms of lanthanide(III) complexes.

Lattice Parameters	PXRD Data Refined by DICVOL06					
	Pr(1)	Sm(2)	Eu(3)	Tb(4)	Dy(5)	Er(6)
System	orthorhombic	orthorhombic	orthorhombic	monoclinic	monoclinic	monoclinic
*a* [Å]	31.9051	32.4828	32.4089	22.9013	22.9579	22.9974
*b* [Å]	25.9574	24.8624	24.9118	7.9797	7.9729	7.9872
*c* [Å]	8.9789	9.2103	9.2154	12.8991	12.8946	12.8856
*α* [°]	90.00	90.00	90.00	90	90	90
*β* [°]	90.00	90.00	90.00	97.223	97.251	97.305
*γ* [°]	90.00	90.00	90.00	90	90	90
V [Å^3^]	7436.09	7438.24	7440.18	2338.55	2341.36	2347.67
M(20)	6.4	5.7	12.5	7.3	8.0	8.4
F(20)	12.1 (0.0115, 144)	14.2 (0.0112, 126)	28.7 (0.0063, 110)	15.9 (0.0161, 78)	14.2 (0.0129, 109)	18.5 (0.0130, 83)

**Table 3:** Thermogravimetric data obtained during heating of lanthanide(III) 1,2-phenylenediacetates in air.

Complex	ΔT_1_ (°C)	Evolved Molecules	Mass Loss (%)		ΔT_2_ (°C)	Mass Loss (%)		Residue
			Found	Calc.		Found	Calc.	
Pr_2_(1,2-pda)_3_(DMF)_2_	120–255	2DMF	15.23	14.55	246–665	66.43	67.3	Pr_6_O_11_
[Sm_2_(1,2-pda)_3_(DMF)_2_]_n_	138–219	DMF	7.5	7.14	-	-	-	-
	220–292	DMF	7.23	7.14	300–803	66.63	66.72	Sm_2_O_3_
[Eu_2_(1,2-pda)_3_(DMF)_2_]_n_	146–208	DMF	7.62	7.12	-	-	-	-
	209–346	DMF	7.34	7.12	350–810	66.96	65.71	Eu_2_O_3_
Tb_2_(1,2-pda)_3_(DMF)_2_	35–323	2DMF	15.23	14.05	325–930	63.67	64.08	Tb_4_O_7_
Dy_2_(1,2-pda)_3_(DMF)_2_	40–327	2DMF	13.2	13.95	328–820 *	63.15	64.4	Dy_2_O_3_
Er_2_(1,2-pda)_3_(DMF)_2_	80–352	2DMF	13.65	13.83	355–850	62.56	63.18	Er_2_O_3_

ΔT_1_—temperature range of desolvation; ΔT_2_—temperature range of degradation of the desolvated form of lanthanide(III) 1,2-phenylenediacetates to suitable oxide; * degradation to oxide occurs with the formation of intermediate Dy_2_O(CO_3_)_2_.

## Data Availability

The data underlying this article will be shared on reasonable request from the corresponding authors.

## Supplementary Materials

The following are available online at https://www.mdpi.com/article/10.3390/ma14174871/s1. Table S1: Selected bonds lengths (Å) and angles (°) for complexes **2** and **3** (coordination environment); Table S2: Selected bonds lengths (Å) and angles (°) for complexes **2** and **3**; Table S3: The hydrogen contacts in structures of complexes 2 and 3; Figure S1: IR spectrum of 1,2-phenylenediacetic acid.
