# Supplementary material for: New Coordination Polymers of Selected Lanthanides with 1,2-Phenylenediacetate Linker: Structures, Thermal and Luminescence Properties
Source: Materials (Basel). 2021 Aug 27;14(17):4871. doi: 10.3390/ma14174871 (PMC8432708; doi:10.3390/ma14174871)
Supplement: Supplementary file 1 [file materials-14-04871-s001.zip › materials-1345840-supplementary.pdf]

Supplementary Material

New coordination polymers of selected lanthanides with 1,2-phenylenediacetate linker: structures, thermal and luminescence properties

Renata Łyszczek[1,*], Iwona Rusinek[1], Agnieszka Ostasz[1], Justyna Sienkiewicz-Gromiuk[1], Dmytro Vlasyuk[1], Marcin Groszek[1], Agnieszka Lipke[2], Oleksiy Pavlyuk[3]

Table S1. Selected bonds lengths(Å) and angles(°) for complexes **2** and **3** (coordination environent).

**2**		**3**	
Sm1-O11	2.391 (7)	Eu1-O11	2.381 (7)
Sm1-O43	2.523 (6)	Eu1-O43	2.496 (7)
Sm1-O44	2.490 (6)	Eu1-O44	2.471 (6)
Sm1-O52	2.357 (6)	Eu1-O52	2.337 (6)
Sm1-O53i	2.509 (6)	Eu1-O53i	2.494 (7)
Sm1-O54i	2.564 (6)	Eu1-O54i	2.560 (7)
Sm1-O61	2.421 (6)	Eu1-O61	2.409 (6)
Sm1-O63i	2.498 (6)	Eu1-O63i	2.485 (6)
Sm1-O64i	2.510 (7)	Eu1-O64i	2.510 (7)
Sm1-C510i	2.897 (9)	Eu1-C510i	2.882 (10)
Sm1-C610i	2.875 (9)	Eu1-C610i	2.864 (10)
Sm2-O13	2.521 (6)	Eu2-O13	2.508 (7)
Sm2-O14	2.470 (7)	Eu2-O14	2.452 (7)
Sm2-O22	2.328 (7)	Eu2-O22	2.311 (7)
Sm2-O23i	2.598 (6)	Eu2-O23i	2.587 (6)
Sm2-O24i	2.523 (6)	Eu2-O24i	2.520 (7)
Sm2-O31	2.494 (7)	Eu2-O31	2.494 (7)
Sm2-O32	2.543 (6)	Eu2-O32	2.537 (7)
Sm2-O33i	2.412 (6)	Eu2-O33i	2.395 (7)

Sm2-O41	2.417 (7)	Eu2-O41	2.410 (7)
Sm2-C210i	2.915 (10)	Eu2-C210i	2.897 (10)
Sm3-O12	2.340 (6)	Eu3-O12	2.331 (6)
Sm3-O13ii	2.494 (6)	Eu3-O13ii	2.478 (7)
Sm3-O21ii	2.380 (6)	Eu3-O21ii	2.374 (6)
Sm3-O23iii	2.411 (6)	Eu3-O23iii	2.395 (6)
Sm3-O31S	2.466 (7)	Eu3-O31S	2.456 (8)
Sm3-O41S	2.460 (7)	Eu3-O41S	2.447 (8)
Sm3-O61	2.485 (6)	Eu3-O61	2.477 (6)
Sm3-O62	2.714 (6)	Eu3-O62	2.718 (7)
Sm3-O63i	2.506 (6)	Eu3-O63i	2.491 (7)
Sm4-O11S	2.440 (7)	Eu4-O11S	2.433 (7)
Sm4-O21S	2.413 (7)	Eu4-O21S	2.405 (7)
Sm4-O32	2.496 (6)	Eu4-O32	2.494 (7)
Sm4-O33i	2.481 (6)	Eu4-O33i	2.482 (6)
Sm4-O34i	2.739 (7)	Eu4-O34i	2.725 (7)
Sm4-O42	2.325 (7)	Eu4-O42	2.320 (7)
Sm4-O44	2.519 (7)	Eu4-O44	2.512 (7)
Sm4-O51	2.394 (6)	Eu4-O51	2.388 (6)
Sm4-O54i	2.442 (6)	Eu4-O54i	2.425 (6)

Table S2. Selected bonds lengths(Å) and angles(°) for **2** and **3.**

Bond lengths (Å)	**2**	**3**
O11-C110	1.241 (12)	1.248 (13)
O11S-C12S	1.234 (12)	1.236 (13)
O12-C110	1.277 (12)	1.260 (12)
O13-C108	1.243 (11)	1.275 (12)
O14-C108	1.262 (11)	1.252 (12)
O21-C208	1.245 (10)	1.262 (11)
O21S-C22S	1.175 (14)	1.185 (14)
O22-C208	1.251 (10)	1.256 (11)

O23-C210	1.277 (10)	1.280 (11)
O24-C210	1.233 (11)	1.247 (12)
O31-C308	1.258 (11)	1.244 (12)
O31S-C32S	1.202 (13)	1.257 (14)
O32-C308	1.284 (11)	1.292 (12)
O33-C310	1.275 (11)	1.289 (11)
O34-C310	1.232 (10)	1.227 (11)
O41-C408	1.233 (12)	1.238 (13)
O41S-C42S	1.234 (12)	1.219 (13)
O42-C408	1.238 (12)	1.249 (13)
O43-C410	1.240 (11)	1.233 (11)
O44-C410	1.268 (12)	1.273 (12)
O51-C508	1.255 (10)	1.264 (11)
O52-C508	1.238 (10)	1.244 (11)
O53-C510	1.233 (10)	1.232 (11)
O54-C510	1.273 (10)	1.285 (11)
O61-C608	1.280 (11)	1.302 (12)
O62-C608	1.238 (10)	1.227 (11)
O63-C610	1.285 (10)	1.293 (11)
O64-C610	1.229 (10)	1.229 (11)
N13S-C12S	1.291 (14)	1.281 (15)
N13S-C14S	1.420 (16)	1.421 (16)
N13S-C15S	1.429 (16)	1.429 (17)
N23S-C22S	1.306 (14)	1.316 (15)
N23S-C24S	1.469 (15)	1.434 (16)
N23S-C25S	1.381 (16)	1.406 (17)
N33S-C32S	1.306 (14)	1.255 (14)
N33S-C35S	1.423 (17)	1.410 (18)
N33S-C34S	1.392 (18)	1.425 (18)
N43S-C42S	1.302 (14)	1.290 (14)
N43S-C44S	1.451 (15)	1.433 (16)
N43S-C45S	1.453 (15)	1.454 (15)
C101-C102	1.401 (12)	1.411 (13)

C101-C106	1.388 (13)	1.385 (13)
C101-C107	1.488 (13)	1.503 (14)
C102-C103	1.392 (13)	1.394 (14)
C102-C109iv	1.516 (13)	1.529 (13)
C103-C104	1.362 (15)	1.367 (16)
C104-C105	1.366 (16)	1.381 (16)
C105-C106	1.376 (15)	1.364 (16)
C107-C108	1.523 (13)	1.516 (14)
C109-C110	1.514 (14)	1.519 (14)
C201-C202	1.386 (12)	1.393 (13)
C201-C206	1.402 (12)	1.391 (13)
C201-C207	1.533 (13)	1.526 (13)
C202-C203	1.379 (13)	1.383 (13)
C202-C209	1.508 (12)	1.513 (12)
C203-C204	1.386 (14)	1.375 (14)
C204-C205	1.347 (15)	1.372 (15)
C205-C206	1.371 (14)	1.371 (15)
C207-C208	1.498 (12)	1.498 (12)
C209-C210	1.507 (12)	1.527 (13)
C301-C302	1.391 (13)	1.396 (14)
C301-C306	1.396 (13)	1.399 (14)
C301-C307	1.487 (12)	1.501 (13)
C302-C303	1.388 (14)	1.387 (15)
C303-C304	1.370 (15)	1.369 (16)
C304-C305	1.378 (15)	1.360 (16)
C305-C306	1.380 (13)	1.390 (14)
C306-C309	1.489 (13)	1.487 (14)
C307-C308	1.502 (12)	1.511 (13)
C309-C310	1.507 (11)	1.515 (12)
C401-C402	1.407 (13)	1.387 (13)
C401-C406	1.377 (15)	1.409 (16)
C401-C407	1.505 (13)	1.508 (14)
C402-C403	1.420 (14)	1.406 (14)

C402-C409	1.492 (13)	1.519 (13)
C403-C404	1.363 (15)	1.372 (16)
C404-C405	1.358 (17)	1.354 (18)
C405-C406	1.381 (16)	1.374 (17)
C407-C408	1.525 (13)	1.526 (14)
C409-C410	1.504 (12)	1.528 (13)
C501-C502	1.374 (12)	1.393 (13)
C501-C506	1.390 (12)	1.389 (12)
C501-C507	1.540 (12)	1.518 (13)
C502-C503	1.371 (13)	1.378 (14)
C502-C509	1.523 (12)	1.517 (12)
C503-C504	1.365 (14)	1.366 (15)
C504-C505	1.366 (14)	1.377 (15)
C505-C506	1.372 (13)	1.387 (14)
C507-C508	1.527 (12)	1.536 (13)
C509-C510	1.486 (12)	1.492 (13)
C601-C602	1.381 (12)	1.411 (13)
C601-C606	1.376 (12)	1.380 (13)
C601-C607	1.490 (12)	1.495 (12)
C602-C603	1.395 (13)	1.383 (14)
C602-C609	1.511 (12)	1.500 (13)
C603-C604	1.363 (15)	1.370 (15)
C604-C605	1.383 (15)	1.380 (16)
C605-C606	1.375 (14)	1.367 (15)
C607-C608	1.499 (13)	1.510 (13)
C609-C610	1.502 (12)	1.514 (13)

Table S3 The hydrogen contacts in structures of **2** and **3.**

D–H···A	D–H	H···A	D···A	D–H···A
C207–H20B...O24[i]	0.97	2.50	3.116(1)	122
	0.97	*2.48*	*3.129(1)*	*124*
C209–H20C...O62[i]	0.97	2.38	3.118(1)	133
	0.97	*2.35*	*3.084(1)*	*132*
C32S–H32S...O61	0.93	2.56	3.150(1)	121
	0.93	*2.58*	*3.153(1)*	*121*
C409–H40D...O42	0.97	2.43	2.999(1)	117
	0.97	*2.39*	*2.975(1)*	*118*
C509–H50D...O34[ii]	0.97	2.32	3.118(1)	140
	0.97	*2.34*	*3.123(1)*	*138*
C607–H60B...O43	0.97	2.48	3.186(1)	129
	0.97	*2.48*	*3.179(1)*	*129*
C206–H206...O22	0.93	2.33	2.970(1)	125
	0.93	*2.34*	*2.975(1)*	*126*
C506–H506...O52	0.93	2.39	3.006(1)	123
	0.93	*2.40*	*3.010(1)*	*123*

Symmetry code: i 1+x,y,z; ii /2-x,y,1/2+z

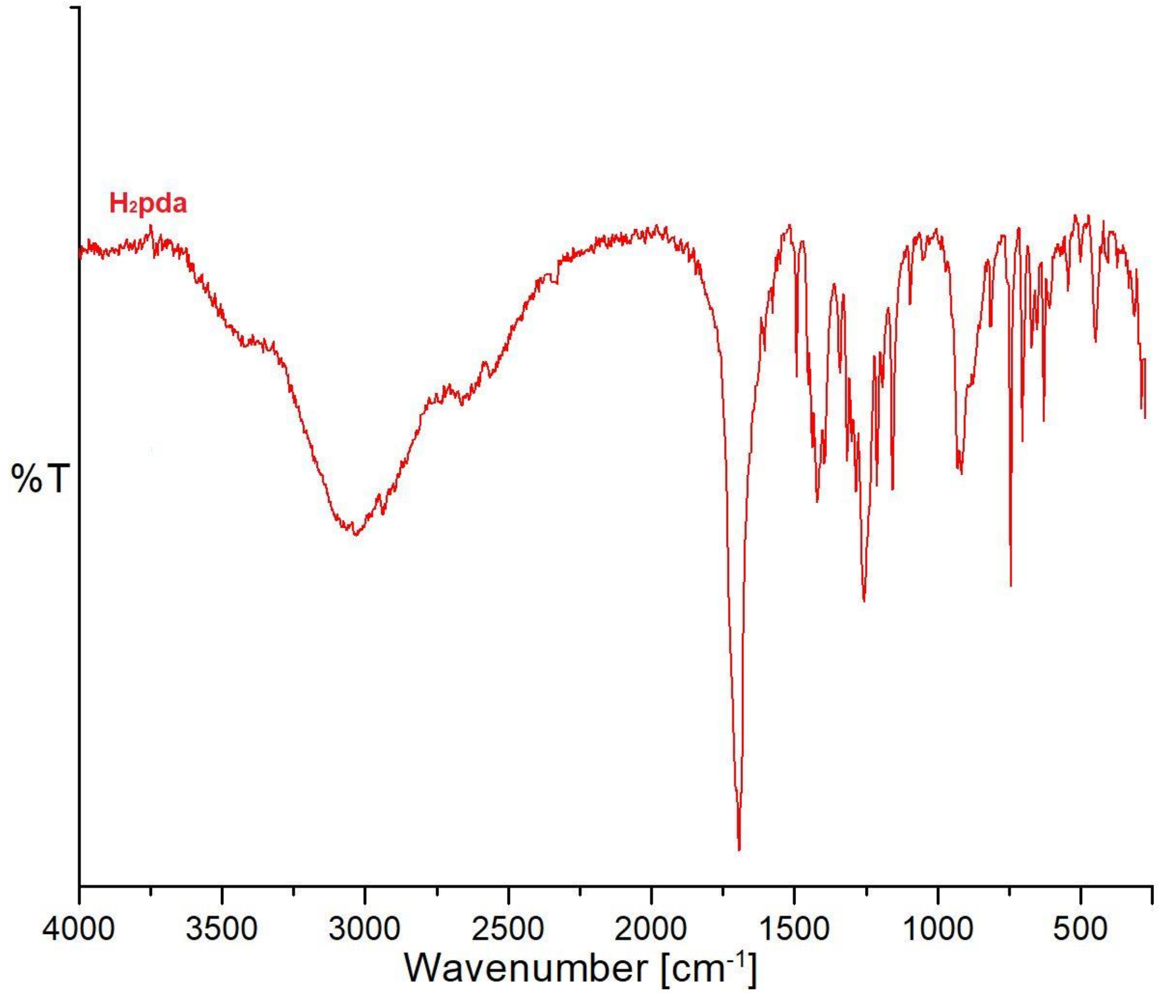

Figure S1. IR spectrum of 1,2-phenylenediacetic acid.
